# Acute Posterior Multifocal Placoid Pigment Epitheliopathy Sharing Characteristic OCT Findings of Vogt-Koyanagi-Harada Disease

**DOI:** 10.1155/2019/9217656

**Published:** 2019-07-09

**Authors:** Yuta Kitamura, Toshiyuki Oshitari, Masayasu Kitahashi, Takayuki Baba, Shuichi Yamamoto

**Affiliations:** ^1^Department of Ophthalmology and Visual Science, Chiba University Graduate School of Medicine, Japan; ^2^Department of Ophthalmology, International University of Health and Welfare, School of Medicine, Japan

## Abstract

A 17-year-old male presented with acute bilateral paracentral scotomata and blurred vision. Funduscopic examination showed bilateral macular serous retinal detachment and yellow-white placoid lesions at the level of retinal pigment epithelium. OCT study showed typical VKH disease findings with marked choroidal thickening and macular serous retinal detachment partly with subretinal septa in both eyes. FA demonstrated hypofluorescence at the placoid lesions in the early phase and hyperfluorescence in the late phase. Laboratory investigation showed negative result for HLA-DR4 serotype and the patient's cerebrospinal fluid test values were within normal range. We made the diagnosis of APMPPE from these results. At 2-month follow-up without the use of corticosteroids, OCT reexamination showed complete amelioration of subretinal fluid in both eyes. Patchy pigmentary lesions also resolved clinically with partial chorioretinal scars. The results in this case suggested OCT findings in APMPPE patients could be similar to characteristic features usually found in acute VKH disease. We recommend comprehensive assessments such as FA, cerebral spinal fluid analysis, and HLA typing which help in leading proper diagnosis.

## 1. Introduction

Acute posterior multifocal placoid pigment epitheliopathy (APMPPE) is a rare inflammatory eye disease typically affecting healthy young adults [1]. Patients typically present with bilateral rapid loss of central vision associated paracentral scotomata. Photopsias may occur prior to vision loss. Central nervous systems disorders such as headache and cerebrovascular vasculitis may be also observed [2]. Approximately 33% of APMPPE patients report flu-like symptoms prior to the onset of the disease [3]. The funduscopic examination typically shows bilateral multiple round, placoid, and gray-white lesions at the level of the retinal pigment epithelium (RPE) with distinctive findings of fluorescein angiography (FA) [1]. Visual outcome is normally good, with more than 80% of APMPPE patients without foveal involvement resolving visual acuity of 20/25 or better, but some patients experienced incomplete restoration of visual function [3]. Therefore, whether treatment should be done or not for the patients with APMPPE is still controversial issue. Although the pathogenesis of APMPPE is still under debate, it is currently thought to be secondary to an obstructive vasculitis, resulting in hypoperfusion of terminal choroidal lobules in the posterior pole with secondary impairment of RPE and retinal outer layer. To our knowledge, three studies have recently reported that APMPPE may share characteristic features with the acute phase of VKH disease, making it difficult to make the diagnosis of two diseases [4–6]. In this paper, we report a case of 17-year-old patient with APMPPE closely resembling OCT findings of acute VKH disease. Because there are still few cases reporting similar contents, we review the latest ocular imaging findings of two diseases and take some key points for proper diagnosis.

## 2. Case Report

**Figure 1:**
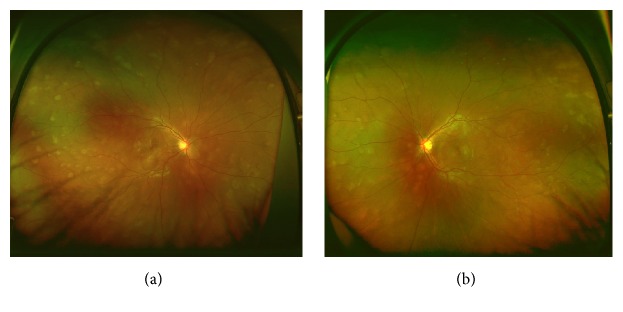
Color fundus photographs with the 200Tx ultra-wide-field retinal imaging system (Optos) at the initial examination demonstrating multiple placoid subretinal lesions scattered from the posterior pole to the peripheral retina bilaterally. Foveal subretinal fluids are present in both eyes. (a) The right eye. (b) The left eye.

**Figure 2:**
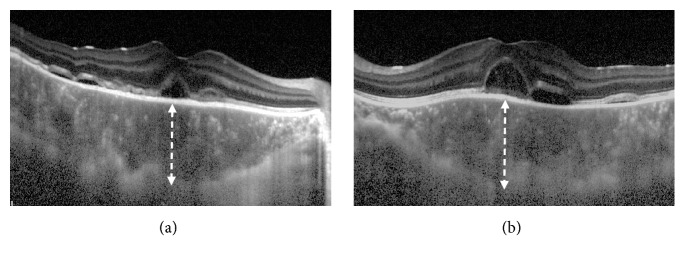
Horizontal enhanced depth optical coherence tomographic (EDI-OCT) images at the initial examination showing marked choroidal thickening and subfoveal detachment with subretinal septa in both eyes. (a) The right eye. (b) The left eye. The choroidal thickness at the subfovea was 578 *μ*m in the right eye and 642 *μ*m in the left eye.

**Figure 3:**
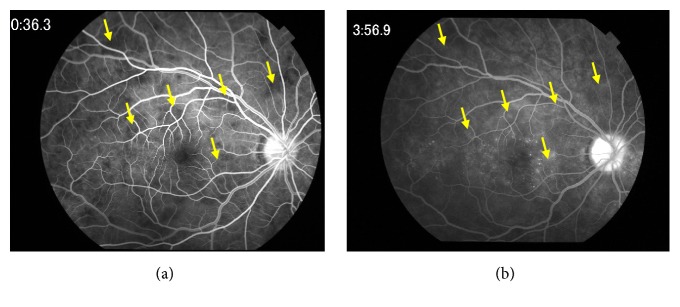
Fluorescein angiography of the right eye at presentation. (a) Early-phase fluorescein angiogram shows areas of hypofluorescence corresponding to the placoid lesions. (b) Late-phase fluorescein angiogram shows the conversion of hypofluorescence to hyperfluorescence (arrows).

**Figure 4:**
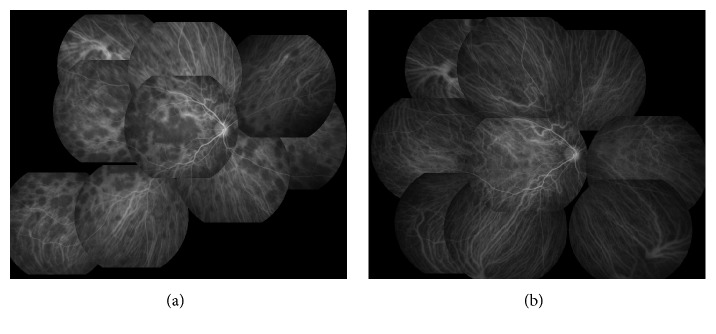
Indocyanine green angiography (ICGA) of the right eye. (a) At presentation showing loss of choroidal circulation with lesions remaining hypofluorescent in the early and late phases and even into the very late phase. (b) At 4 months after the initial examination. ICGA shows an almost complete resolution of the choroidal hypofluorescence.

**Figure 5:**
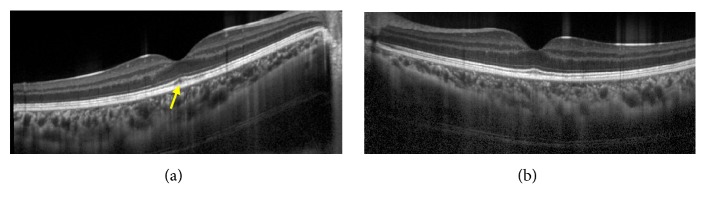
Horizontal EDI-OCT images of the right (a) and left (b) eyes at 2 months after the initial presentation. Subretinal fluid disappeared completely and choroidal thickness recovered within normal range in both eyes. The external limiting membrane and ellipsoid zone (inner/outer segment junction) and retinal pigment epithelium are clearly recognized bilaterally, but there are unclear parts in the interdigitation zone (arrow). The choroidal thickness at the subfovea was 300 *μ*m in the right eye and 275 *μ*m in the left eye.

A 17-year-old male high school student was referred to Chiba University Hospital because of rapid bilateral paracentral scotomata and blurred vision on awaking. He complained from a viral prodrome such as headache and nasal discharge 1 week before the onset of visual disturbance, although there were no symptoms of hearing loss or tinnitus. Medical history and family history were unremarkable. He took no medication. On examination, best-corrected visual acuity was 20/16 in both eyes. Intraocular pressure was normal. There were no light reflex disorders in both eyes. The ocular movements were normal. Slit-lamp examination showed 1+ anterior chamber cells in both eyes. Funduscopic examination showed bilateral macular serous retinal detachment, and the one-fourth to one-second disc diameter, placoid, multiple round, and yellow-white lesions at the level of RPE from posterior pole to far peripheral retina (Figure 1). The placoid lesions were more obvious in the peripheral retina rather than posterior pole. The retinal vessels and optic disc were normal. Spectral domain OCT (SD-OCT) showed marked choroidal thickening and macular serous retinal detachment partly with subretinal septa in both eyes, which are known as characteristic OCT findings in VKH disease (Figure 2). FA of the both eyes revealed that the placoid lesions were hypofluorescence during early-phase FA images and subsequent progressive hyperfluorescence on late-phase FA images (Figure 3). Fundus autofluorescence (FAF) revealed the lesions were observed as increased fluorescence corresponding to the placoid lesions. Indocyanine green angiography (ICGA) revealed that the lesions were hypocyanescent in the early and late phases of the ICGA (Figure 4(a)). Visual fields analysis using by the Humphrey field analyzer 30-2 test showed bilateral paracentral scotomata. Laboratory investigation showed the absence of the HLA-DR4 serotype. Cerebrospinal fluid (CSF) examination was also normal. The laboratory screening was negative results for any known infectious or autoimmune cause. We made the diagnosis of APMPPE on the basis of these data. Because of good vision and absence of systemic neurological abnormalities, we did not administer oral prednisone. Instead, the patient was instructed to take Ibuprofen orally. At 1-month follow-up, OCT showed bilateral decrease of subretinal fluid. At 2 months' follow-up, OCT showed complete resolution of subretinal fluid and decrease of choroidal thickness in both eyes (Figure 5). Four months after the initial presentation, Patchy pigmentary lesions resolved clinically partly with chorioretinal scars and hypofluorescence lesions on ICGA images showed improvement in clinical findings (Figure 4(b)). There has been no recurrence until now.

## 3. Discussion

This case highlights the importance of detecting characteristic FA findings, absence of HLA-DR4, and absence of CSF lymphocytic pleocytosis to differentiate between APMPPE and VKH disease because, in some cases, APMPPE disease patients share some common clinical manifestations such as preceding viral prodrome, morphological changes on OCT such as subretinal fluid with multilobular spaces, marked choroidal thickening, and ICGA imaging study.

APMPPE is basically diagnosed with the characteristic fundus finding of multiple placoid yellow-white creamy deep retinal lesions, the typical pattern of early hypofluorescence and late hyperfluorescence corresponding to the placoid lesions on the FA images, and prolonged hypocyanescence on the ICGA imaging study [7]. Typical OCT findings observed in the APMPPE patients are slight irregularities in the inner segment ellipsoid layer and RPE, at early phase, subsequently evolving increased reflectance of the outer retinal layers and RPE with normal retinal thickness, and at later phase the thinning of the outer retina layer gradually occurs. Subretinal fluid may be observed in the APMPPE patients associated with large placoid lesions. [7]

Whereas the diagnosis of acute VKH disease is mainly based on typical fundus findings and OCT images, patient's history of preceding syndrome such as flu-like symptoms, headache, dysacusia, and tinnitus, lumbar puncture, and HLA typing are also useful diagnostic tests. CSF evaluation reveals lymphocytic pleocytosis in more than 80% of the VKH patients. HLA-DR4 is known to have strong association among Japanese patients with VKH syndrome. In acute phase of VKH disease, characteristic enhanced depth imaging OCT (EDI-OCT) features include multilobular serous retinal fluid, marked choroidal thickening, and choroidal folds (CFs) without pigment epithelial detachment [8]. The exact nature of serous detachment and septa was studied by Kishi et al. [9, 10], and they suggested the walls of cystoid spaces are made of inflammatory products, such as fibrin, and are located in the subretinal space. In acute phase of the disease, FA typically reveals a large number of spotted hyperfluorescent foci at the level of RPE in the early stage, followed by patchy pooling of dye in the subretinal space. ICGA study discloses an early choroidal stromal vessel hyperfluorescence and leakage, disc hyperfluorescence, multiple hypofluorescent dark, and fuzzy or lost pattern of large stromal vessel [11]. Among these imaging studies, OCT findings of characteristic exudate pooling with septa and marked choroidal thickening have been considered to highly valuable diagnostic findings associated with acute VKH disease.

In this case, the manifestations that teenage patient, with preceding flu-like symptoms, had acute onset of creamy multiple placoid deep retinal lesions, classic FA findings of “block early, stain late” angiographic pattern corresponding to the placoid lesions, persistent patchy hypocyanescence on the ICGA study, and negative findings of CSF lymphocytic pleocytosis and HLA-DR4 clearly suggest diagnosis of APMPPE. However, the most distinguishing feature in this case is that the patient demonstrated marked choroidal thickening and macular serous retinal detachment with subretinal septa on OCT imaging, not typical features in APMPPE but quite similar to characteristic features in acute VKH disease. According to a classification of the OCT findings observed in the APMPPE patients suggested by Goldenberg et al. [12], subretinal fluid have been reported at the very acute phase of the disease, but the nature of subretinal fluid and the frequency of septa formation are still controversial. In contrast with typical findings seen in VKH patients, choroidal thickening is not a typical OCT finding in patients with APMPPE. However, Lee et al. firstly reported this finding in the literature [6], and, more recently, two studies reported thickening imaging with EDI-OCT has been observed in APMPPE patients [13, 14].

Since the initial description by Gass in 1968, APMPPE was presumed to involve primarily RPE [1], whereas several researchers suggested that the underlying process in APMPPE is a focal inflammatory vasculitis affecting the choroidal vessels and that the RPE changes were a subsequent manifestation [15–17]. Recently, by using laser speckle flowgraphy (LSFG), Hirotaka et al. showed that the choroidal blood flow velocity decreased at the acute stage of APMPPE and subsequently increased with regression of the disease [13]. Moreover, with the advent of OCT-angiography (OCT-A), many researchers have noted choriocapillaris hypoperfusion, preceding the development of and corresponding anatomically to placoid lesions in the RPE [18, 19]. Bruke et al. proposed four characteristic sequential phases of APMPPE lesions by using multimodal imaging study including ten eyes of five APMPPE patients [7]. They concluded that choriocapillaris hypoperfusion is the primary event in the pathogenesis of APMPPE. These circulatory changes and choroidal thickening on EDI-OCT provided further evidence that inflammatory etiology was involved in the pathogenesis of choroidal circulatory disturbance in APMPPE [20]. From these viewpoints, anti-inflammatory agents such as systemic corticosteroid, intravitreal injection of triamcinolone acetonide have been used to treat APMPPE patients. In our case, subretinal fluid and choroidal thickening resolved spontaneously without taking oral corticosteroids, suggesting that there might be an unknown self-limit inflammatory process in the choroid.

Consistent with past reports, our patient showed the RPE line maintained almost flat on SD-OCT at the acute stage of disease although marked choroidal thickening was observed. In contrast, in 52-71% of VKH patients, choroidal folds (CFs), which are wave-like appearance in the RPE, Bruch's membrane and inner choroid, have been found on SD-OCT [21–23]. The recent study indicated that detection of both CFs and choroidal thickening is an effective method for diagnosing acute VKH disease [24]. However, another recent report has shown that the folds of RPE occurred less frequently in younger patients with VKH patients [25]. Therefore, in young patients, the lack of CFs on SD-OCT cannot clearly differentiate APMPPE from VKH. The reason why older patients with VKH disease developed CFs more frequently is still unknown.

As shown in this case, the choroidal hypofluorescence of ICGA image in eyes with APMPPE has been reported to result from a profound decrease in choroidal blood flow secondary to choroidal occlusive vasculitis [26, 27]. In VKH disease, one of the ICGA signs is known as multiple hypofluorescent spots, which are thought to correspond to foci of lymphocytic infiltration within the choroid. The hypofluorescent lesions detected by ICGA may persist even after the fundoscopic examination and FA are unremarkable [28, 29]. These reports indicate that ICGA findings of VKH disease may be similar to those of APMPPE because both show multiple hypofluorescent lesions.

In summary, we have reported some key points of distinguishing APMPPE from VKH.

In some cases, APMPPE and VKH disease may share some common clinical manifestations such as preceding viral prodrome and several imaging findings, which are morphological changes on OCT such as subretinal fluid, choroidal thickening, flat line of RPE (especially younger patients), and ICGA imaging study. Therefore, it is important for ophthalmologist to detect characteristic FA findings and HLA typing and investigate CSF evaluation to differentiate these two diseases.
